# Hyperfibrinolysis after parapelvic cyst surgery: A case report

**DOI:** 10.3892/etm.2012.795

**Published:** 2012-11-02

**Authors:** CHUN-HUA TANG, LI-JING SHEN, QIANG GAO, YI YANG, LI-XING CHEN

**Affiliations:** 1 Department of Urology, Songjiang Hospital Affliated to First Hospital of Shanghai, Songjiang;; 2 Department of Hematology, Renji Hospital, Shanghai Jiaotong University School of Medicine, Shanghai, P.R. China

**Keywords:** parapelvic cyst, hyperfibrinolysis, diagnosis and treatment

## Abstract

The present study describes the diagnosis and treatment of hyperfibrinolysis following surgery in a 25-year-old female patient. An examination revealed that the left kidney had been affected by severe hydronephrosis for two weeks prior to hospitalization. The diagnosis of a parapelvic cyst was obtained by preoperative intravenous pyelogram (IVP), computed tomography (CT) and upper left urinary tract retrograde pyelography. Unroofing of the left parapelvic cyst was performed by open surgery. The patient exhibited symptoms of shock 48 h later, and her hemoglobin (Hb) levels dropped to only 62.2 g/l. To treat this, 400 ml erythrocyte suspension transfusion was administered 3 times every other day. The patient’s Hb levels remained between 50 and 60 g/l. The D-dimer assay index rose from 0.3 to 16 mg/l and the fibrin degradation product (FDP) levels progressively increased following the hemorrhage, while the platelet count, prothrombin time (PT), activated partial thromboplastin time (APTT) and fibrinogen (Fg) index were all within normal levels. p-Aminomethylbenzoic acid (PAMBA; 0.5 g) was administered to the patient every day, and as a consequence the Hb levels rose steadily from the next day onwards. After a one week course of PAMBA treatment, the patient’s condition became stable. Blood coagulation and fibrinolytic function measurements were all within the normal ranges in the three months following the surgery. Delayed hemorrhage following surgery should be considered as a possible cause of hyperfibrinolysis. Monitoring FDP and D-dimer levels may aid a rapid and clear diagnosis. Anti-fibrinolytic therapy, such as PAMBA treatment, is safe and effective for use against the type of hemorrhage caused by hyperfibrinolysis.

## Introduction

Hyperfibrinolysis is a condition in which the natural ability to dissolve blood clots is pathologically enhanced. Hyperfibrinolysis occurs more frequently following primary diseases, including severe trauma, liver surgery, organ transplantation and obstetrical accidents, may cause hemorrhagic manifestations of varying severity. It is present in 2–8% of trauma patients and associated with shock and increased mortality (1). No previous domestic or foreign literature has reported hyperfibrinolysis following common urological surgery. Our hospital recently reported the following case.

## Case report

**Figure 1:**
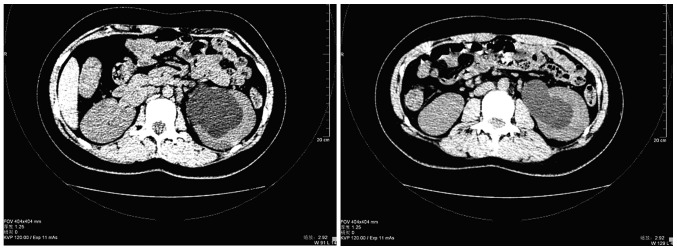
Different computed tomography (CT) scans of the abdomen prior to surgery. This revealed left hydronephrosis and a parapelvic cyst, with no visible stones in the middle and distal segments of the double ureter, and little fluid accumulation in the pelvic cavity.

**Figure 2:**
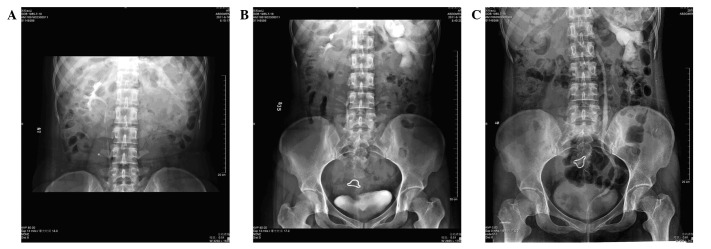
Upper urinary tract retrograde pyelography (RGP) plus intravenous pyelogram (IVP) prior to surgery. IVP and RGP revealed left hydronephrosis and a parapelvic cyst, with the left renal calyces markedly dilated. (A) Left renal secretion function had been damaged. (B) Left renal excretion function had been damaged. (C) Parapelvic cyst had occupied the whole renal pelvis.

A 25-year-old female was admitted to Shanghai Songjiang Hospital in June 2011 with severe hydronephrosis in the left kidney. Her past medical history was unremarkable. Physical examination revealed soft percussion pain in the area of the left kidney. The patient’s bilateral ureter and bladder region demonstrated no sign of tenderness, and her body temperature was normal. All blood counts and biochemical data were normal. Computed tomography (CT) of the abdomen revealed left hydronephrosis and a parapelvic cyst, with no visible stones in the middle or distal segments of both ureters and little fluid accumulated in the pelvic cavity (Fig. 1). An upper urinary tract retrograde pyelography plus intravenous pyelogram (IVP) also revealed left hydronephrosis and a parapelvic cyst (Fig. 1), with markedly dilated left renal calyces (Fig. 2). A diagnosis of a left parapelvic cyst was then made. On July 5th 2011, an unroofing surgery of the left parapelvic cyst was successfully performed under general anesthesia in 1.5 h. The patient was safely escorted to the common ward.

**Figure 3:**
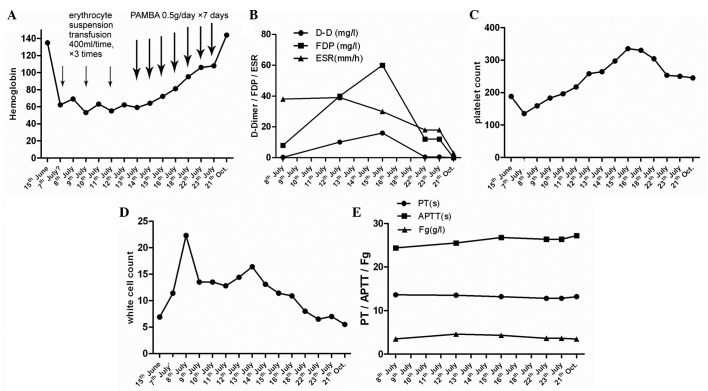
Blood coagulation and fibrinolytic data statistics of clinical course. (A) The change of hemoglobin. Hemoglobin levels increased gradually after PAMBA therapy. (B) The changes of D-dimer and FDP, which increased sharply after hemorrhage and decreased synchronously when hemoglobin improved. (C, D and E) The white cell and platelet count, PT, APTT and Fg index remained at normal levels. PAMBA, p-aminomethylbenzoic acid; FDP, fibrin degradation product; ESR, erythrocyte sedimentation rate; PT, prothrombin time; APTT, activated partial thromboplastin time; Fg, fibrinogen.

On the night of July 7th (48 h post-surgery), the patient developed a progressive heart rate, was pale and sweating, and her blood pressure dropped to 85/50 mmHg. The immediate peripheral blood cell counts revealed a hemoglobin (Hb) level of 62 g/l, a white cell count of 11,400/μl and a platelet (PLT) count of 135,000/μl (Fig. 3). The patient was administered an immediate transfusion of 400 ml erythrocyte suspension and was infused with appropriate fluids. The next day the patient’s blood pressure and heart rate became stable again. Further laboratory tests showed that the prothrombin time (PT) was 13.6 sec, the thrombin time (TT) was 15 sec, the activated partial thromboplastin time (APTT) was 24.4 sec, the D-dimer assay recorded 0.3 mg/l and the fibrin degradation product (FDP) level was 8 mg/l. The patient received further erythrocyte suspension transfusions of 400 ml on July 9th and 11th. The Hb level did not improve (range, 53–69 g/l), but the PLT count returned to normal (159,000–258,000/μl) afterwards.

**Figure 4:**
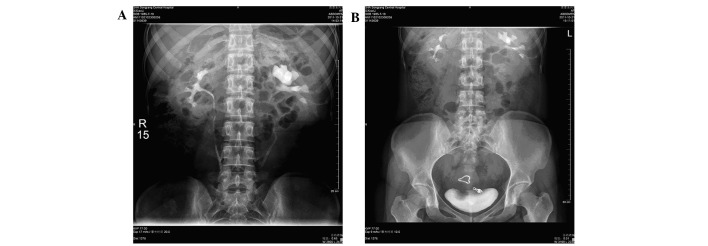
Follow-up IVP examination three months post-surgery. The left hydronephrosis and parapelvic cyst were resolved. (A) Left renal secretion function has improved. (B) Left renal excretion function has improved. IVP, intravenous pyelogram.

On July 12th, a CT scan of the abdomen revealed an increase in left kidney volume, a renal hemorrhage and a small number of blood clots around the left kidney. PT was 13.5 sec, APTT was 25.5 sec and fibrinogen (Fg) levels were 4.6 g/l, all within normal ranges. However, the D-dimer assay result was 10.18 mg/l and the FDP level was 40 mg/l, both of which had notably increased. Considering the existence of hyperfibrinolysis, we treated the patient with 0.5 g p-aminomethylbenzoic acid (PAMBA) per day from July 13th. The patient’s Hb levels steadily increased and reached 72 g/l (Table I) in two days. The patient was consequently discharged from hospital in a good condition 10 days later. A follow-up IVP examination was performed three months post-surgery (Fig. 4); complete blood cell counts, blood coagulation and fibrinolytic function measurements were all within normal ranges. This case report was approved by the ethics committee of the Songjiang Hospital Affliated to First Hospital of Shanghai, and informed patient consent was obtained.

## Discussion

### Activation and functions of fibrinolysis

The main functions of the fibrinolytic system in the body are to remove fibrin deposition, dissolve blood clots in the vessel walls and maintain a smooth bloodstream. The system mainly consists of plasminogen (PLG), plasminogen activator (PA), plasminogen activator-specific inhibitors (PAIs), plasmin and plasmin inhibitor. The fibrinolytic system may be activated by the coagulation process or directly by fibrinolytic enzymes which are induced by certain factors. A key factor in the triggering of the fibrinolytic process, tissue-type PA (t-PA), exists in a variety of organs, including the uterus, ovary, prostate, lung and kidney. The stress response, shock, malignancies, organ transplantation, trauma and thrombolytic drugs also affect the t-PA activity.

### Types of hyperfibrinolysis

Hyperfibrinolysis is abnormally enhanced activity in the fibrinolytic system, resulting in premature fibrin, excessive damage and/or the degradation of blood coagulation factors, including fibrinogen, thus causing bleeding. There are two kinds of hyperfibrinolysis, congenital and acquired. Congenital hyperfibrinolysis is extremely rare. Only 10 cases have been reported in the literature to date. Clinical hyperfibrinolysis cases are mainly acquired (1). Acquired hyperfibrinolysis has two types, primary and secondary. Primary acquired hyperfibrinolysis is a hemorrhagic syndrome caused by certain primary diseases which induce the increase of t-PA, kallikrein and activating factor XII (FXIIa), or the decrease of fibrinolysis system inhibitors such as PAI. Secondary acquired hyperfibrinolysis is a thrombosis-hemorrhage syndrome induced by certain primary diseases which cause local or disseminated intravascular coagulation (DIC) or hypercoagulative states, intravascular fibrin precipitates and the release of t-PA into the blood circulation.

### Diagnosis of hyperfibrinolysis

The main clinical manifestation of hyperfibrinolysis is hemorrhage but there is a lack of other specific symptoms. Patients may exhibit spontaneous skin ecchymosis, mucosal bleeding, surgery or post-traumatic wound site and injection site bleeding and bleeding of joints and muscles, GI tract, urinary tract and catheter insertion sites. In addition, certain patients have the symptoms of the primary disease.

FDP and D-dimer assays are the essential indices of fibrinolytic activity (2). FDP assays count the total amount of X, Y, D and E fragments. These are the products of plasmin-induced fibrin and reflect the overall level of fibrinolytic activity, which increases in both primary and secondary hyperfibrinolysis. A D-dimer is a specific degradation product formed by FXIIa from cross-linked fibrin monomers, followed by plasmin hydrolysis. Healthy individuals have D-dimer levels <500 ng/ml, while the D-dimer levels of patients with different types of thrombotic diseases or hypercoagulative states (following surgery or pregnancy) can reach several thousand nanograms per milliliter, depending on the activity of fibrinolysis. Monitoring the D-dimer level aids the identification of primary and secondary hyperfibrinolysis. The direct detection of PA, PAI, Fg and PLG may reflect the cause of fibrinolysis more concretely, however, few hospitals to date have carried out such a comprehensive examination.

Secondary hyperfibrinolysis is mostly caused by DIC, which is always accompanied by increased FDP and D-dimer levels, although these two indices rise slightly in a variety of physiological hypercoagulation situations. According to the British Journal of Hematology DIC Diagnosis and Treatment Guidelines (3), abnormal DIC laboratory test results are obtained with decreasing probability in this order; PLT decreased, FDP increased, PT prolonged, APTT prolonged and Fg decreased. APTT and PT, the conventional indicators of coagulation factors, measure the intrinsic and extrinsic coagulation function, respectively, although they are not sensitive and only work with sufficient coagulation factor consumption (>20–30%). However, if PLT count, Fg level and plasma protamine paracoagualtion tests (3P tests) are taken into consideration at the same time, it should not be difficult to diagnose DIC at an early stage.

### Therapy of hyperfibrinolysis

Currently, fibrinolytic therapy includes the application of a range of fibrinolytic inhibitors and blood transfusion products. ε-aminocaproic acid (EACA), tranexamic acid and PAMBA are drugs that are able to competitively inhibit the combination of plasminogen and fibrin, so that plasminogen is not activated to become plasmin, thus achieving an anti-fibrinolytic function. These drugs are effective in patients suffering from hemorrhage caused by severe liver disease or surgery. Tranexamic acid and PAMBA are ∼10 and 4–5 times more potent, respectively, than EACA. The CRASH-2 Joint Working Group (4) holds the opinion that such drugs should be used as soon as possible in patients with severe trauma.

Aprotinin is a natural serine protease inhibitor that is able to irreversibly combine and inactivate a variety of serine proteases, including fibrinolytic enzymes and kallikrein. Aprotinin is also able to affect multiple intermediates, thus resulting in the attenuation of inflammatory responses, the inhibition of primary and secondary fibrinolysis and thrombin generation. There have been a number of studies (5–7) on the application of aprotinin in liver transplants and cardiothoracic surgery. The normal intake of this drug is 80,000–100,000 units every day, in 2 to 3 doses. The main side-effects are anaphylaxis and renal dysfunction, which are particularly likely to occur in patients who receive a second application within six months.

Fibrinogen is a protein synthesized by the liver which has functions in blood coagulation and is also the precursor of fibrin. Thrombin converts fibrinogen into fibrin monomers. When these monomers cross-link with each other, they become a stable insoluble protein fiber clot to complete the coagulation process. Patients with severe liver function disorders or severe trauma, whose plasma fibrinogen concentrations have decreased, may have a hemorrhagic tendency. Generally, a 2 g Fg infusion increases the blood plasma Fg level by 0.5 g/l. When Fg levels reach 1.25 g/l or above, the hemorrhaging stops (8).

A new type of dynamic analytical instrument, the thromboelastography (TEG) coagulation analyzer, is now in clinical application, and being used to monitor the blood coagulation process. The Rotational Thrombosis Elastic Measurement (ROTEM) derived from the TEG is able to fully reflect the process in patients, from blood coagulation to fibrinolysis, and has achieved good results on guiding the treatment of trauma patients (9,10).

### Experience of diagnosis and treatment

Our patient exhibited symptoms of shock 48 h post-surgery. PLT count, PT, APTT and Fg tests were performed multiple times and the results were all within the normal range; the cause of the symptoms was therefore considered to be chronic blood loss, which may be controlled by routine hemostasis and erythrocyte suspension transfusions. However, after nearly one week of active treatment, the patient’s Hb level demonstrated no improvement. The FDP and D-dimer levels progressively increased, while PLT count, PT and APTT remained within the normal ranges. With no evidence of DIC, we believed that the hypercoagulative state of the patient was due to chronic blood loss and t-PA released into the blood circulation from the kidney following surgery. This activated the fibrinolytic enzymes directly and thus induced the secondary hyperfibrinolysis and caused a hemorrhage syndrome. While FDP has a strong anticoagulant effect, it aggravated the hemorrhage and worsened the patient’s situation.

As an established drug that is safe and effective, PAMBA was administered promptly as the antifibrinolytic treatment. A good result was achieved as the patient recovered quickly.

### Conclusion

The main lessons we have learnt from this case arose during diagnosis. The patient had hemorrhage symptoms 48 h post-surgery, and following consideration of the PLT count, PT, APTT, FDP, D-dimer assay and Fg test results, which were were all within the normal ranges at the time, we only administered a blood transfusion and anti-hypovolemia treatment to the patient. We did not realize that a severe hypercoagulative state would induce hyperfibrinolysis symptoms, so an FDP or D-dimer assay test was not performed in time. This was, therefore, the main reason that an effective treatment result was not obtained at the beginning of treatment. We did not identify the rising FDP and D-dimer levels until 6 days had passed since the hemorrhage symptoms occurred. We were fortunate that there were no serious consequences.

The key to curing hyperfibrinolysis patients lies in whether the primary disease or predisposing factors can be dealt with quickly. In this case, the hyperfibrinolysis was induced subsequent to surgery, without evidence of therioma or DIC. An effective result was achieved by antifibrinolytic treatment.

In conclusion, for future patients with delayed hemorrhage after surgery or non-effective blood transfusion, we shall consider the possibility of hyperfibrinolysis. We will monitor the FDP, PT and APTT levels to ensure that we diagnose the disease in time, prior to using a safe and effective therapy to cure the patient.

## Figures and Tables

**Table I:** Laboratory test indices and therapy.

	Date (day/month)													
Index	15/06	07/07	08/07	09/07	10/07	11/07	12/07	13/07	14/07	15/07	16/07	18/07	22/07	21/10
WBC (×10^9^)	6.9	11.4	22.3	13.5	13.5	12.8	14.4	16.4	13.1	11.4	10.9	8.0	6.5	7.0
ANC%	53	81	92.5	77.2	77.1	73.2	72.7	72.7	73.2	75.1	75.9	77.1	66.1	58.8
RBC (×10^12^)	4.6	2.14	2.36	1.93	2.17	1.86	2.1	2.03	2.22	2.49	2.4	2.54	2.83	4.83
Hb (g/l)	135	62.2	69.2	53.2	63.2	55.2	62.2	59.2	64.2	72.2	71.2	75.2	86	144
PLT (×10^9^)	188	135	159	183	196	217	258	264	297	335	330	304	253	245
PT (sec)			13.6				13.5			13.2			12.8	13.2
Fg (g/l)			3.48				4.6			4.32			3.63	3.45
APTT (sec)			24.4				25.5			26.8			26.4	27.2
D-D (mg/l)			0.3				10.18			16			0.5	neg
FDP (mg/l)			8				40			60			12	neg
ESR (mm/h)			38				39			30			18	3
RBC suspension transfusion (units)		2		2		2								
PAMBA (g)								0.5	0.5	0.5	0.5	0.5		

WBC, white blood cell; ANC, absolute neutrophil count; RBC, red blood cell; Hb, hemoglobin; PLT, platelet; PT, prothrombin time; Fg, fibrinogen; APTT, activated partial thromboplastin time; D-D, D-dimer; FDP, fibrin degradation product; ESR, erthythrocyte sedimentation rate; PAMBA, p-aminomethylbenzoic acid; neg, negative.
